# A Treatise on the Principles and Practice of Medicine

**Published:** 1886-12

**Authors:** 


A Treatise on the Principles and Practice of Medicine. Designed for the use of Practitioners and Students of Medicine. *By* Austin Flint, M.D., LL.D., *late Professor of the Principles and Practice of Medicine, and of Clinical Medicine, in the Bellevue Hospital Medical College, etc. Sixth Edition, revised and largely rewritten by the author, assisted by* William H. Welch, M.D., *Professor of Pathology in Johns Hopkins University, and* Austin Flint, M.D., LL.D., *Professor of Physiology in the Bellevue Hospital Medical College. pp. 1,160. Philadelphia:* Lea Brothers & Company, *1886. Chicago:* A. C. McClurg & Company.

It will be a great satisfaction to the admirers of the late Professor Austin Flint, Sr., to know that this edition of his "Practice of Medicine" was revised under his own immediate supervision. Dr. William H. Welch has written the first seven chapters and a large part of the eighth in Part I, and he has also revised and rewritten the description of anatomical characters of the diseases considered in the rest of the volume.

Special attention is called in the preface to the following entirely new articles: Infectious Tumors; Syphilitic Disease of the Lungs; Cerebral Syphilis; General Considerations relating to Inflammatory and Structural Diseases of the Spinal Cord; Spastic Cerebral Paralysis of Children; Hereditary Ataxia; Myxœdema; Multiple Neuritis; General Pathology of Fever and Milk Sickness; "and in nearly every article changes and additions, some of them very important, have been made."

The statement is made that this edition "contains a full consideration of recent discoveries concerning the bacterial origin of various infectious diseases." With Dr. Welch as sponsor for this statement it may be accepted without question.

Five diagrammatic engravings have been introduced in connection with the nervous system. "By careful condensation and rearrangement, and by the rigid omission of obsolete matter, the additions contained in this edition have been accommodated without an increase in the number of pages."

The limits of a mere book notice do not allow a critical consideration of the new matter introduced, and the reputation of the author and his assistant render such consideration unnecesary. The book may be truly considered "the rcowning work of his (the author's) long professional life." It may be said to more fairly "represent the existing state of medical science, with respect to those subjects of which it treats," than any other English work. Those physicians who have the fifth edition will want the sixth, and those who have neither ought to have the last.

